# Increased Mortality in Metal-on-Metal versus Non-Metal-on-Metal Primary Total Hip Arthroplasty at 10 Years and Longer Follow-Up: A Systematic Review and Meta-Analysis

**DOI:** 10.1371/journal.pone.0156051

**Published:** 2016-06-13

**Authors:** B. G. Pijls, J. M. T. A. Meessen, J. W. Schoones, M. Fiocco, H. J. L. van der Heide, A. Sedrakyan, R. G. H. H. Nelissen

**Affiliations:** 1 Department of Orthopaedics, Leiden University Medical Center, Leiden, The Netherlands; 2 Department of Medical Statistics, Leiden University Medical Center, Leiden, The Netherlands; 3 Mathematical Institute, Leiden University, Leiden, The Netherlands; 4 Walaeus Library, Leiden University Medical Centre, Leiden, The Netherlands; 5 FDA Medical Device Epidemiology (MDEpiNet) Science and Infrastructure Center, U.S. Food and Drug Administration, Silver Spring, MD, United States of America; 6 Department of Healthcare Policy and Research, Weill Cornell Medical College, New York, NY, United States of America; Glasgow University, UNITED KINGDOM

## Abstract

**Importance:**

There are concerns about increased mortality in patients with metal-on-metal bearings in total hip arthroplasty (THA).

**Objective:**

To determine the mortality and the morbidity in patients with metal-on-metal articulations (MOM THA) compared to patients with non-metal-on-metal articulations (non-MOM THA) after primary total hip arthroplasty.

**Data Sources:**

Search of PubMed, MEDLINE, EMBASE, Web of Science, Cochrane, CINAHL, AcademicSearchPremier, ScienceDirect, Wiley and clinical trial registers through March 2015, augmented by a hand search of references from the included articles. No language restrictions were applied.

**Study Selection:**

Two reviewers screened and identified randomised controlled trials and observational studies of primary total hip arthroplasty comparing MOM THA with non-MOM THA.

**Data Extraction and Synthesis:**

Two reviewers independently extracted study data and assessed risk of bias. Risk differences (RD) were calculated with random effect models. Meta-regression was used to explore modifying factors.

**Main Outcomes and Measures:**

Difference in mortality and difference in morbidity expressed as revisions and medical complications between patients with MOM THA and non-MOM THA.

**Results:**

Forty-seven studies were included, comprising 4,000 THA in randomised trials and over 500,000 THA in observational studies. For mortality, random effects analysis revealed a higher pooled RD of 0.7%, 95%, confidence interval (CI) [0.0%, 2.3%], I-square 42%; the heterogeneity was explained by differences in follow-up. When restricted to studies with long term follow-up (i.e. 10 years or more), the RD for mortality was 8.5%, 95%, CI [5.8%, 11.2%]; number needed to treat was 12. Further subgroup analyses and meta-regression random effects models revealed no evidence for other moderator variables (study level covariates, e.g. resurfacing vs. non-resurfacing MOM) than follow-up duration. The quality of the evidence presented in this meta-analysis was characterized as moderate according to the CLEAR-NPT (for non-pharmacological trials) and Cochrane risk of bias Table.

**Conclusions and Relevance:**

Meta-analysis suggests there may be an increased long-term risk of mortality and revision surgery for patients with MOM THA compared to patients with non-MOM THA.

**Registration:**

PROSPERO 2014:CRD42014007417

## Introduction

Metal-on-metal bearings have been used since the early years of total hip arthroplasty (THA) development, and are still used today with 2.000 procedures in 2014 in the National Joint Registry alone [1]. Early historical prostheses from the 1960's 1970's and 1980s include the McKee Farrar hip and Ring hip prosthesis [2]. They can be considered the first generation of metal-on-metal total hip arthroplasty (MOM-THA). However, a recent long-term follow-up study of first generation MOM-THA reported increased mortality in patients with metal-on-metal bearings in total hip arthroplasty compared to patients with non-metal-on-metal bearings [3]. While this is an isolated report, metal-on-metal bearings in total hip arthroplasty are known to produce metallic particles due to wear and corrosion [4]. These metallic particles may lead to local and systemic adverse effects (e.g. nephrotoxicity, cardiotoxicity, carcinogenicity, and structural changes in the visual pathways and basal ganglia), which in turn could lead to increased mortality [5, 6]. These reports are in conflict with two recent registry-based studies of modern, second generation MOM-THA which do not report higher mortality associated with metal-on-metal hips [7, 8]. However, there are concerns that registry-based studies in this setting may be subject to residual confounding [9].

The purpose of this systematic review and meta-analysis is therefore to determine the overall mortality and morbidity in randomised controlled trials and observational studies for first- and second-generation metal-on-metal bearings compared to non-metal-on-metal bearings after primary total hip arthroplasty in patients with endstage primary and secondary osteoarthritis.

## Materials and Methods

The reporting of this systematic review is in accordance with the PRISMA statement and a protocol has been registered a priori at the Prospero registry (PROSPERO 2014:CRD42014007417) S1 and S2 Files.[10]. After the PROSPERO protocol was registered, we also performed a systematic review of observational studies evaluating mortality and medical complications (i.e. cancer incidence, kidney failure or cardiomyopathy) for metal-on-metal bearings compared to non-metal-on-metal bearings in patients with total hip arthroplasty. This would allow us to compare the results from randomised controlled trials (RCTs) to the results from observational studies. The population of interest consisted of patients treated with primary total hip arthroplasty due to endstage primary and secondary osteoarthritis of the hip after failed conservative treatment. The intervention group consisted of patients who received metal-on-metal bearings, including total hip resurfacing with metal bearings: MOM THA. The control group consisted of patients with primary total hip arthroplasty with non-metal-on-metal bearings (e.g. metal-on-polyethylene, metal-on-ceramic, ceramic-on-ceramic, ceramic-on-polyethylene): non-MOM THA.

The primary outcome was mortality, expressed as the number of patients who died within the study period. The secondary outcome was morbidity, expressed as the number of surgical and medical complications experienced by the subjects within the study period.

### Data Sources and Searches

The search strategy was composed in collaboration with a librarian experienced in the field of total hip arthroplasty, and included studies, abstracts, and trial registry records from the date of their their inception to the end of March 2015. The following databases were searched: PubMed, MEDLINE, EMBASE, Web of Science, Cochrane, CINAHL, Academic Search Premier. The following journal publisher databases were also searched: ScienceDirect and Wiley. References of included articles were screened for relevant studies. Finally, clinical trial registers (clinicaltrails.org; WHO InternationalClinicalTrialsRegistryPlatform; Multi-register; DutchTrialRegistry) were searched to identify any ongoing trials that were completed but not yet published. Contact persons of eligible trial registry records were contacted by e-mail, and at least two reminders were sent in case of no response.

The search strategy for the RCTs consisted of the following components, each defined by a combination of controlled vocabulary and free text terms:

1. implant type: metal-on-metal, resurfacing and brand names
2. total hip arthroplasty
3. randomised controlled trial.

An example Pubmed search is provided in S3 File.

### Study Selection

Initially, the literature was screened on title and abstract. This screening was performed by two reviewers (BP and JM) independently. Both reviewers recorded their findings in a pre-designed electronic database. Both databases were then compared and any disagreements were resolved by consensus or by consulting a referee. When the information in the abstract did not suffice, or if any doubt remained, the studies remained eligible.

The fulltext papers of eligible studies were independently evaluated by two reviewers (BP and JM). Both recorded their findings in a pre-designed electronic database. Any disagreements were resolved by consensus or by consulting a referee.

All bibliographic records identified through the electronic searches were collected in an electronic reference database and subjected to the following inclusion and exclusion criteria:

**Inclusion criteria:** 1) primary total hip arthroplasty

2) comparison of metal-on-metal bearing with non-metal–on-metal bearing

3) randomised controlled trial or quasi-randomised controlled trial (for RCTs)

4) follow-up of at least 3 months.

**Exclusion criteria:** 1) only bilateral cases with metal-on-metal and non-metal-on-metal in the same patient (this would not allow us to determine mortality for the groups separately)

2) no reporting/evaluation of mortality or morbidity.

### Data Extraction and Quality Assessment

Two reviewers (BP and JM) independently extracted data and appraised the risk of bias from included studies regarding mortality and morbidity, patient demographics, study characteristics, and implant specifications in a pre-defined electronic data sheet. The data sheet was designed during the extraction of trial data on a random sample of eligible studies. Any disagreements were resolved by consensus or by consulting a referee.

Risk of bias was appraised at the level outcome using the CLEAR-NPT checklist and Cochrane risk of bias table [11]. The CLEAR-NPT checklist was specifically designed to appraise the methodological quality of non-pharmacological trials and contains items related to the standardization of the intervention, care provider influence, and additional measures to minimize the potential bias from lack of blinding of participants, care providers, and outcome assessors [11]. Any disagreements were resolved by consensus or by consulting a referee.

### Data Synthesis and Analysis

A random effects model was employed to pool the risk difference of individual studies in order to estimate an overall risk difference and its associated confidence interval. The inverse variance method, which gives more weight to larger studies, was used to pool outcomes for different studies. The overall effects, corresponding to a random effects model, is reported in the forest plots along with its confidence intervals. The sizes of the square boxes on the forest plots are proportional to the total number of patients in the selected studies. An overall test on heterogeneity between studies was performed. To estimate between-study variance, DerSimonian-Laird’s method was employed [12]. In case moderators are incorporated in the model, the weighted estimation gives an estimate of the weighted least squares relationship between the moderator variables and the true effect. All analyses were performed using Metafor Package R statistics [13]. The measure of interest chosen was risk difference (RD) to account for any "empty cells" for mortality or morbidity corresponding to a particular study.

Randomised controlled trials of first and second generation MOM THA and observational studies of first generation MOM THA (evolution of prosthesis development) were eligible for meta-analysis. Observational studies of second generation MOM were considered subject to strong selection bias [7–9], so they were not eligible for meta-analysis. The amount of heterogeneity was assessed through visual inspection of forest plots and by calculating tau-squared statistics (which is the amount of heterogeneity in the true RDs) and I-squared statistics. The latter estimates how much of the total variability in the effect size estimates is due to heterogeneity among the true effects. In the presence of heterogeneity, and if data allowed, random effects meta-regression on pre-defined factors (study level covariates) was employed. These factors were defined in the PROSPERO protocol: type of metal bearing (resurfacing vs. non-resurfacing), type of non-metal bearing, head size, fixation method (cemented vs. cementless), indication for THA (primary vs. secondary osteoarthritis), methodological items from CLEAR NPT and Cochrane risk of bias Table, duration of follow-up, mean age at operation, gender distribution (% of females and males), and pre-operative health (American Society of Anesthesiologist (ASA) scores).

To assess for publication bias, we constructed a funnel plot for studies reporting the primary outcome. In the case of asymmetry in the funnel plot, or if publication bias was suspected based on the trial registries, a trim-and-fill method and cumulative meta-analysis was used to explore the magnitude and direction of publication bias.

## Results

### RCTs

**Fig 1:**
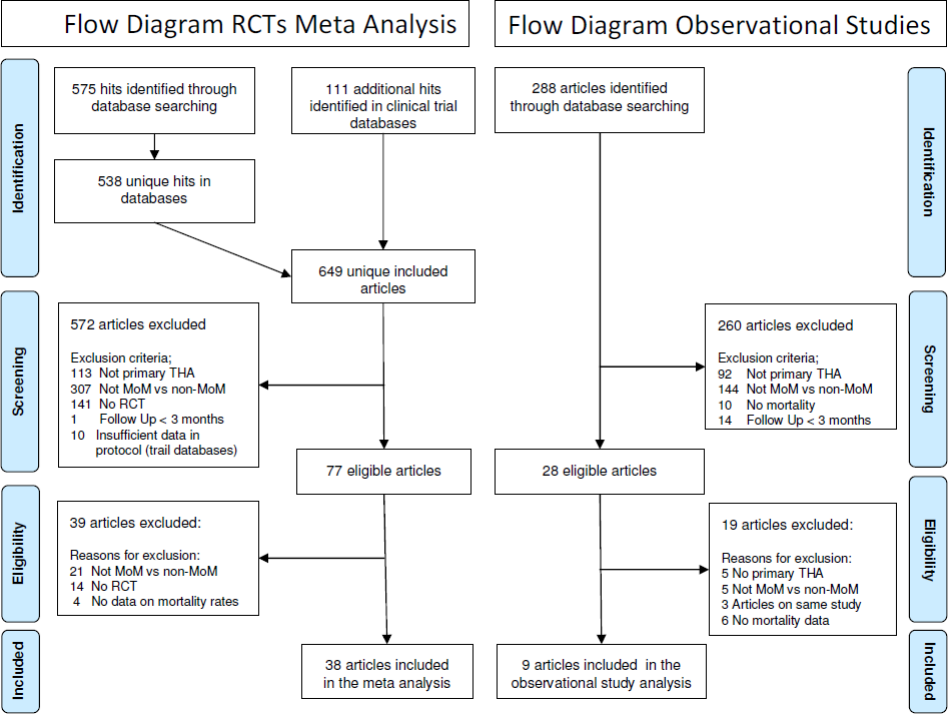
PRISMA flow chart.

The literature search yielded 686 hits and 30 studies (38 papers) published between 1975 and 2014 were included, for a total of 1,806 patients with MOM THA and 2,151 patients with non-MOM THA [2, 14–42]. Three studies were not published in peer reviewed journals (1 abstract, 2 trial registry reports) [19, 21, 41] and 27 studies [2, 14–18, 20, 22–40, 42] were published in 38 papers; 7 studies on the same RCT were published in more than one paper, including1 study that was published in 3 papers. These papers were mostly follow-up reports. For the analyses, we used the paper with the longest follow-up. Details of study selection and flow of the review are shown in Fig 1 and details of included studies are shown in Table 1.

**Table 1:** Details of included RCTs.

Author	Publication	MoM						non-MoM					followup time
	year	Generation	N patients	N hips	N deaths	N revision	Resurfacing	N patients	N hips	N deaths	N revision	type non-MoM	(year)
Zagra	2013	2nd	20	20	0	0	no	40	40	0	0	Polyethylene—Ceramic	0,33
Grubl	2006	2nd	15	15	0	0	no	13	13	0	0	Ceramic—Ceramic	1
Schouten	2012	2nd	39	39	0	0	no	42	42	0	0	Ceramic—metal	1
Jensen	2011	2nd	21	21	0	0	yes	22	22	0	0	Polyethylene—Ceramic	1
Zijlstra	2011	2nd	25	25	0	0	no	25	25	0	0	Polyethylene—metal	1
Hanna	2012	2nd	28	28	0	0	no	23	23	0	0	Polyethylene—metal	2
Weissinger	2011	2nd	42	42	0	.	no	38	38	0	.	Ceramic—Ceramic	2
Tiusanen	2013	2nd	46	46	0	2	no	46	46	0	1	Polyethylene—metal	2
Penny	2012	2nd	21	21	0	0	yes	22	22	0	0	Polyethylene—metal	2
nct00208494		2nd	196	196	1	.	no	194	194	1	.	Ceramic—metal	2
Gauthier	2013	2nd	25	25	0	1	no	25	25	2	0	Polyethylene—metal	2
Malviya	2011	2nd	50	50	1	2	no	50	50	2	2	Polyethylene—metal	4
Brodner	2003	2nd	50	50	1	1	no	50	50	2	1	Polyethylene—Ceramic	5
Gustafson	2014	2nd	26	26	0	1	yes	26	26	0	0	Polyethylene—metal	5
Macdonald	2005	2nd	23	23	1	0	no	18	18	0	0	Polyethylene—metal	5,1
Wang	2012	2nd	37	37	0	0	yes	40	40	0	0	Ceramic—Ceramic	6
Engh	2014	2nd	68	68	2	1	no	37	37	2	1	Polyethylene—metal	6,5
Bjorgul	2013	2nd	123	129	6	8	no	251	268	16	4	mixed	7
Zerahn	2011	2nd	74	74	6	.	no	225	225	14	.	mixed	7,6
nct01422564		2nd	12	12	2	.	no	12	12	0	.	Polyethylene—metal	8
Hailer	2011	2nd	41	41	1	.	no	44	44	4	.	Polyethylene—metal	8
Howie	2005	2nd	11	11	0	8	yes	13	13	0	2	Polyethylene—metal	10
Desmarchelier	2013	2nd	111	125	19	3	no	116	125	13	1	Ceramic—Ceramic	10
Zijlstra	2010	2nd	101	102	30	4	no	97	98	23	2	Polyethylene—metal	10,7
Pabinger	2003	2nd	31	32	1	1	no	28	29	1	0	Polyethylene—Ceramic	.

The search of the trial registry reports yielded 111 hits, of which 12 were deemed eligible. The contact persons of these 12 trials were approached. Four did not respond, even after at least 2 reminders. Eight did respond, which resulted in the inclusion of 2 trials. One additional trial was already included as a journal version. Five trial registry reports were excluded because the study was not a randomised controlled trial (n = 4) or there was no information available on mortality or morbidity (n = 1).

### Observational studies

The literature search yielded 288 hits and 9 studies were included, with a total of 78,110 patients with MOM THA and 451,605 patients with non-MOM THA, published between 1996 and 2014 [3, 7, 8, 43–48]. Details of study selection and flow of the review are shown in Fig 1 and details of included studies are shown in Table 2.

**Table 2:** Results from observational studies.

Outcome	Study	Resurfacing	MOM generation	IRR	CI	FU	n MOM	n non-MOM
All cause	Visuri 2010	No	1st	1.05	0.95–1.16	17	579	1585
Mortality								
	Lubbeke 2014	No	2nd	0.90*	0.70–1.20	9,6	883	2458
	Makela 2014	Mixed	2nd	0.78	0.69–0.88	4,6	10728	18235
	McMinn 2012a	Yes	2nd	0.61*	0.50–0.75	3,6	8352	53409
	McMinn 2012b	Yes	2nd	0.68*	0.55–0.84	3,6	8352	50529
	Kendal 2013a	Yes	2nd	0.51*	0.45–0.59	6	7437	22311
	Kendal 2013b	Yes	2nd	0.55*	0.47–0.65	5	8101	24303
								
Cancer	Visuri 2010	No	1st	1.27	0.98–1.63	17	579	1585
Mortality								
	Makela 2014	Mixed	2nd	0.78	0.63–0.97	4,6	10728	18235
								
Cardiac Mortality	Visuri 2010	No	1st	1.07	0.93–1.22	17	579	1585
	Makela 2014	Mixed	2nd	0.79	0.64–0.97	4,6	10728	18235
								
Cancer incidence	Visuri 1996	No	1st	1.25	0.99–1.58	13,5	698	1831
	Smith 2012	No	2nd	1.02*	0.93–1.12	3	21264	248995
	Lamohamed 2013	No	2nd	1.04*	0.70–1.56	3,2	988	9714
	Makela 2012	Mixed	2nd	0.92	0.81–1.05	4	10728	18235
	Smith 2012	Yes	2nd	0.72*	0.61–0.86	3	19312	248995

* hazard ratio. a = cemented. b = uncemented. IRR = incidence rate ratio. FU = follow up in years.

### Mortality

There were 25 RCTs (31 papers) that reported mortality [14, 16–27, 29, 31–38, 40–42]. These RCTs comprised 1,225 patients with MOM THA (71 mortalities) and 1,486 patients with non-MOM THA (80 mortalities). There were five observational studies that reported mortality: one with first generation MOM THA and four with second generation MOM THA [3, 7, 8, 43, 48].

**Fig 2:**
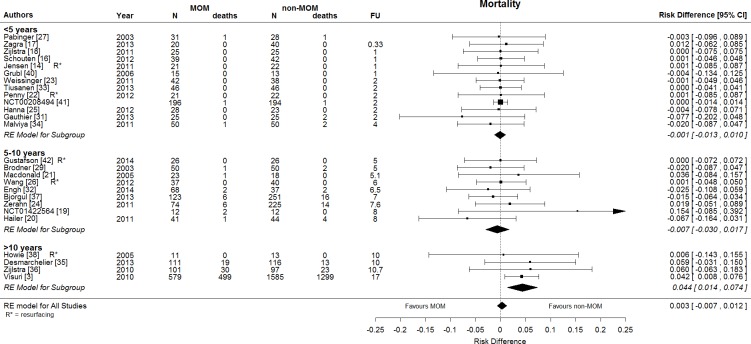
Forest plot showing the risk difference in mortality between MOM THA and non-MOM THA. Overall, there is no difference in mortality between MOM THA and non-MOM THA. After 10 years, there is an increased risk of mortality for MOM THA. Besides one observational study [3] of first generation MOM all other studies are RCTs. RE model = random effects model.

Meta-analysis of RCTs and first generation MOM observational studies [3] showed a difference trend towards higher mortality for MOM THA: RD 0.7%, 95% CI [0.0%, 2.3%], I-square equal to 42%. Fig 2 shows the results of three different meta-analyses, including the RD of the MOM vs. non-MOM studies and the 95% CI associated to each individual study. The overall effect for each separate meta-analysis based on a random effects model is shown. This heterogeneity, I-square 42%, was explained by differences in follow-up, as shown in Fig 2. After correction for follow-up with random effects meta-regression, there was no residual heterogeneity, and I-square was equal to 0%.

When restricted to studies with long term follow-up (10 years or more) [3, 35, 36, 38], the RD was 8.5%, 95% CI [5.8%, 11.2%]; number needed to treat was 12. This analysis used the unadjusted data from Visuri et al. [3] When using adjusted data from Visuri et al [3], the RD was equal to 4.4%, 95% CI [1.4%, 7.4%]. Further subgroup analyses and meta-regression revealed no evidence for other modifying factors (e.g. resurfacing vs. non-resurfacing MOM). Sensitivity analyses with “leave one out” methodology indicated that the results were not significantly influenced by any single study.

Table 2 shows all-cause mortality and cause-specific mortality for first and second generation MOM observational studies. The first generation MOM observational study, which looked at non-resurfacing MOM in patients with primary osteoarthritis, showed a trend towards increased risk of mortality for patients with MOM compared to non-MOM THA [3], Incidence Rate Ratio 1.05, which is in line with the long term results from the RCTs. The second generation MOM observational studies showed decreased risk of mortality for patients with MOM compared to non-MOM-THA, Hazard Ratios ranging from 0.51 to 0.90 [7, 8, 43, 48]. This is in contrast with the long-term results from RCTs and first generation observational study.

### Morbidity: surgical complications

**Fig 3:**
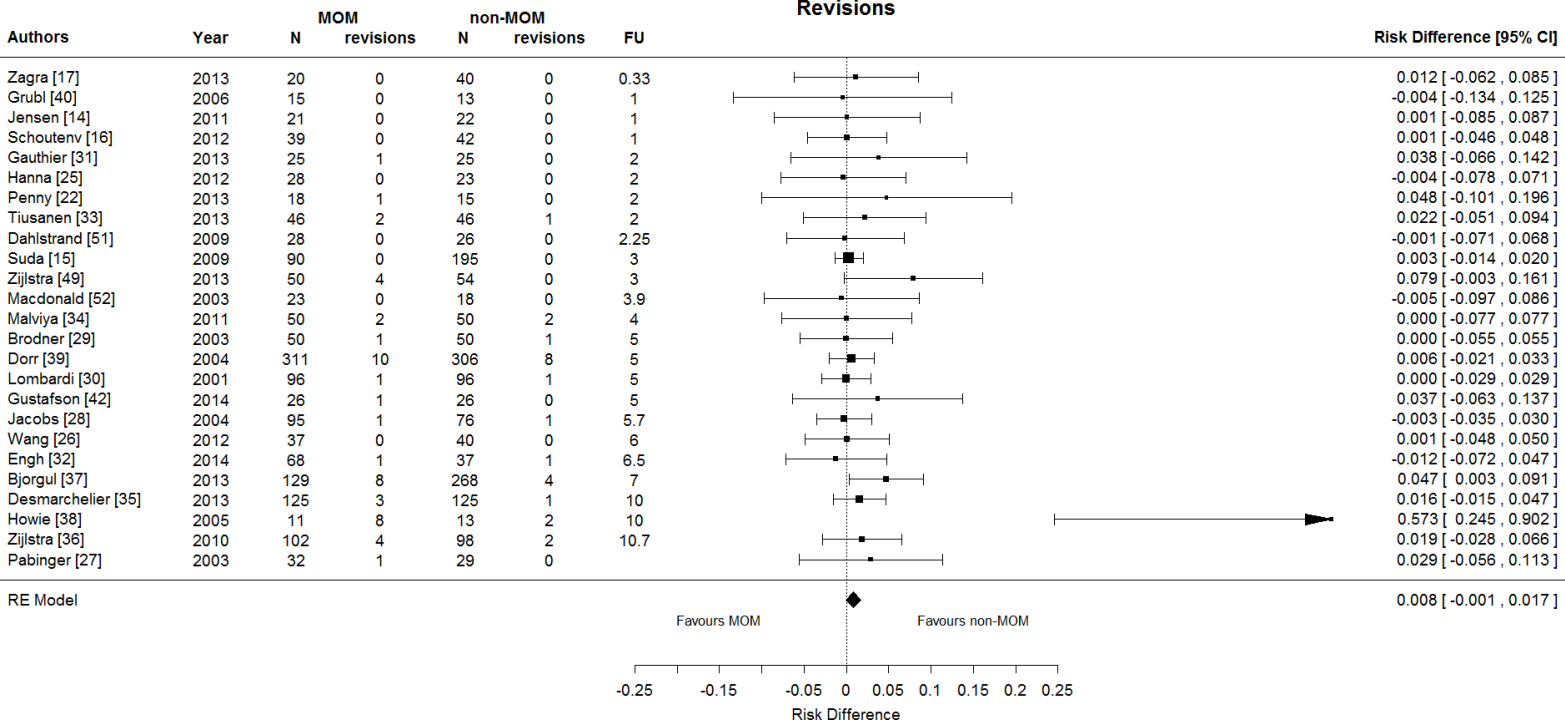
Forest plot showing the risk difference in revision surgery between MOM and non-MOM THA. There is significantly more revision surgery for MOM THA compared to non-MOM THA. RE model = random effects model.

There were 26 RCTs (30 papers), all of second generation MOM THA, that reported revisions [14–17, 25–40, 42, 49–52]. These studies comprised 1,546 MOM THA (49 revisions) and 1,746 non-MOM THA (24 revisions). There were more revisions in MOM THA compared to non-MOM THA: RD 0.8%, 95% CI [-0.1%, 1.7%]; I-square 0%; random effects meta-analysis presented in Fig 3. This effect was stronger for cemented THA, with more revisions in MOM than non-MOM THA: RD 2.7%, 95% CI [0.1%, 5.3%]; number needed to treat was 37.

Regarding revision for aseptic loosening the RD was 0.6%, 95% CI [-0.3%, 1.4%], and regarding revision for septic loosening the RD was 0.3%, 95% CI [-0.3%, 0.9%].

Sensitivity analyses with “leave one out” methodology indicated that the results were not significantly influenced by any single study.

### Morbidity: medical complications

There were four RCTs, all of second generation MOM THA, that reported medical complications, with maximum follow-up ranging from 2 to 10 years [19, 35, 41, 50]. Since there were only three or fewer RCTs that reported on each medical complication (nephrotoxicity, cardiotoxicity, carcinogenicity, and general medical complications [e.g. venous thrombosis]) meta-analysis was not considered appropriate. Data from single studies are reported in Table 3.

**Table 3:** Medical complications for RCTs.

Author	Year	MOM					non-MOM				
		N patients	N cancer	N nephro	N cardio	N general	N patients	N cancer	N nephro	N cardio	N general
NCT00208494		196	2	1	4	35	194	3	3	5	37
NCT01422564		12	1	.	1	.	12	0	.	0	.
Desmarchelier	2013	111	.	.	.	2	116	.	.	.	5
Penny	2013	18	.	.	.	1	15	.	.	.	0

nephro = nephrotoxicity. cardio = cardiotoxicity.

There were four observational studies that reported cancer incidence: one with first generation MOM THA and three with second generation MOM THA, see Table 2 [44–47]. The first generation MOM observational study showed increased risk of cancer for patients with MOM compared to non-MOM THA [44]. The second generation MOM observational studies showed no difference in risk of cancer for patients with MOM compared to non-MOM THA [45–47].

### Risk of bias

**Fig 4:**
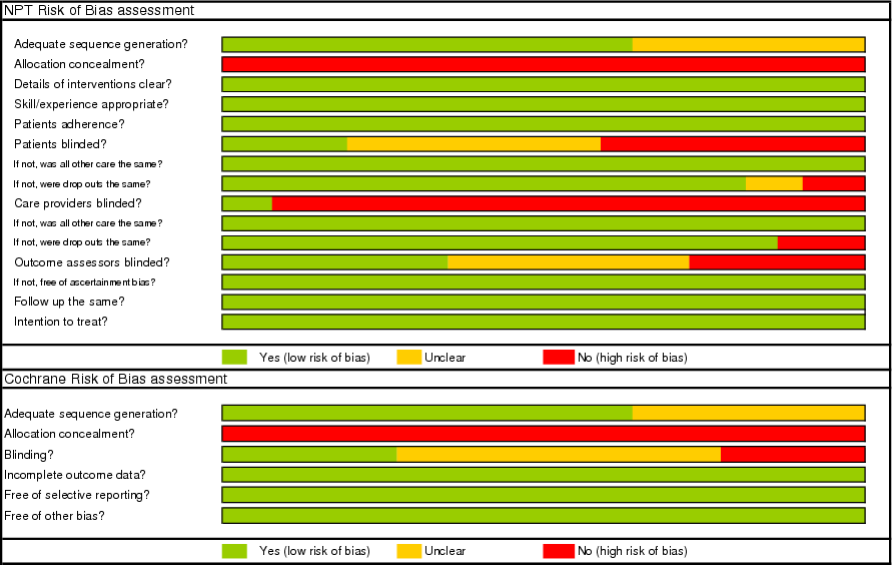
Risk of bias tables for CLEAR NPT and Cochrane.

Risk of bias items from the CLEAR-NPT and Cochrane are presented in Fig 4. All studies suffered from problems with allocation concealment and blinding of patients, care givers, and outcome assessors.

The strong points of all studies were that compliance with the treatment was of course 100%, follow-up was similar for both MOM and non-MOM groups, and the skill/experience of the surgeons was similar for MOM and non-MOM THA (non-resurfacing).

The results from observational studies of second generation MOM THA were different from those of first generation MOM THA and those of the RCTs, suggesting strong confounding by indication for observational studies of second generation MOM THA [9].

### Publication bias

**Fig 5:**
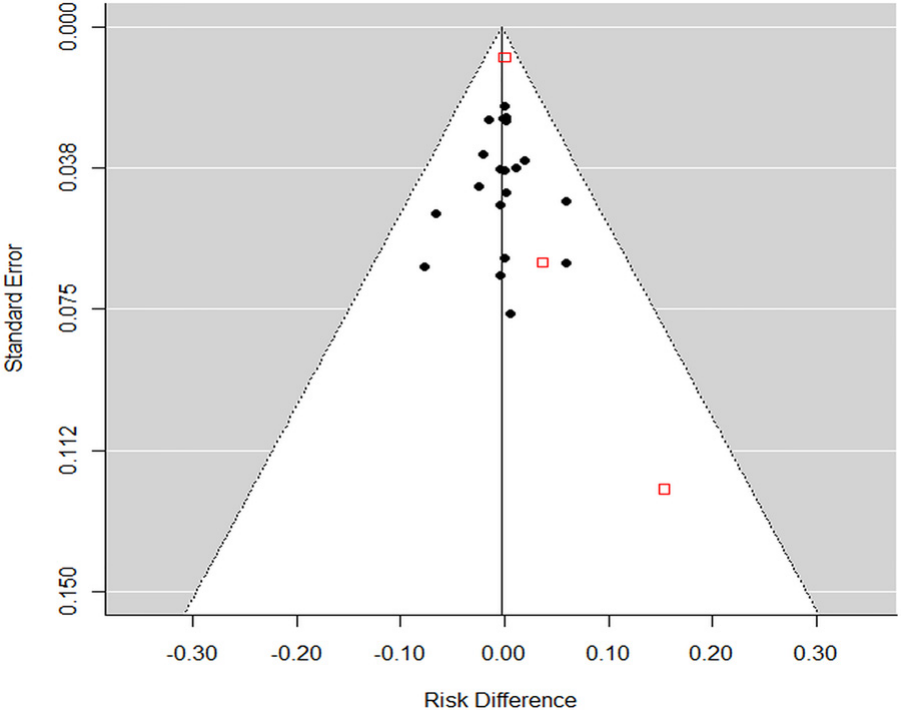
Funnel plot of RCTs. The red open boxes represent 1 abstract and 2 trial registry reports that have not been published.

The potential influence of publication bias is small, as shown by a nearly symmetrical funnel plot in Fig 5. Also, the trim-and-fit method and the cumulative meta-analysis showed small potential influence of publication bias that would not influence the results. Furthermore, the results from the non-published RCTs (identified from the trial registries) were similar to those of published studies: RD for mortality in the non-published studies was -0.3%, 95% CI [-1.8%, 1.1%], and in the published studies was 0.1%, 95% CI [-1.3%, 1.5%].

## Discussion

### Principal findings

We found that when restricting to studies with long term follow-up (10 years and more), [3, 35, 36, 38] there was an increased risk of mortality in patients with MOM THA compared to patients with non-MOM THA: RD 8.5%, 95% CI [5.8%, 11.2%]. This finding, compared to a lack of difference between MOM and non-MOM THA patients with less than 10 years’ follow-up, might indicate a dose-response association. The longer patients are exposed to MOM THA, the higher the risk of mortality is compared to non-MOM THA. Importantly, sensitivity analyses with meta-regression showed that duration of follow-up was the only effect modifier.

Regarding surgical morbidity, there were more revisions in MOM THA compared to non-MOM THA: RD 0.8%, 95% CI [-0.1%, 1.7%], based on 26 RCTs of second generation MOM THA. When restricted to cemented THA, this effect was stronger: RD 2.7%, 95% CI [0.1%, 5.3%].

Since data on post-operative medical complications were reported in only a few studies, no valid meta-analysis could be done on differences between the two THA groups. Regarding the observational studies, one first generation MOM study showed an increased risk of cancer for MOM patients compared to non-MOM THA patients[44]. The second generation MOM observational studies showed no difference in overall cancer risk. However, risk of soft-tissue sarcoma and basalioma was higher for MOM THA patients [48].

The risk of mortality for MOM THA from observational studies of second generation MOM THA was different from those of first generation MOM THA and RCTs between (non)MoM THA, suggesting confounding by indication in studies of second generation MOM THA as previously reported by Kandala et al [9].

In a recent review, Hartmann et al demonstrated that metal ion concentrations were persistently elevated after implantation of MOM bearings in whole blood, serum, plasma, erythrocytes and urine, irrespective of patient characteristics and study characteristics [6]. Of concern is that the same authors found very high serum cobalt concentrations in several of their included studies—above 50 μg/L, while the detection limit for serum cobalt is typically 0.3 μg/L. They found the highest metal ion concentrations in patients with a stemmed, large-head MOM implant and in patients with hip resurfacing arthroplasty. Our sensitivity analyses did not identify any association between MOM head size (either resurfacing or THA) and mortality or surgical complications. However the number (25) and size (2700 pts) of our included RCTs may have been too small to detect a difference.

### Toxic and carcinogenic effects

Devlin et al and Bradberry et al have shown in a systematic review that patients with suspected Prosthetic Hip Associated Cobalt Toxicity (PHACT) had symptoms that fell in three categories: neuro-ocular toxicity, cardiotoxicity and thyroid toxicity [53, 54]. The signs and symptoms developed between 3 and 72 months (median 19 months) after the MOM THA, [53, 55]. The most common treatment of PHACT in literature was removal of the metal-containing prosthesis, which resulted in lowered cobalt concentration and improvement of symptoms [53–55]. Of great concern is also the fact that the International Agency for Research on Cancer (IARC) has classified cobalt as group 2B, *Possibly carcinogenic to humans"* [56]. Furthermore, Moulin et al have shown that metal workers exposed to cobalt have an increased mortality rate from lung cancer [57].

Although most emphasis in literature is on cobalt toxicity (PHACT), the effects of chronic exposure to elevated chromium or nickel levels should not be dismissed. The International Agency for Research on Cancer (IARC) has classified chromium and nickel as group 1, "*carcinogenic to humans"* [56]. Chromium(VI) in particular is carcinogenic through direct DNA damage after intra-cellular reduction to chromium(III), mutation, genomic instability, aneuploidy, and cell transformation [56]. Exposure to chromium by ingestion or inhalation is associated with increased risk of lung cancer, sinonasal cancer, and stomach cancer [56, 58–61]. The connection between chromium inhalation/ingestion and an increased risk of lung cancer, sinonasal cancer, stomach cancer, and possibly melanoma do not directly extrapolate to increased cancer risk due to increased plasma chromium levels in MOM THA. Briggs et al have shown a strong relationship between whole blood levels of chromium and total chromosomal aberration indices in peripheral lymphocytes of MOM patients. [62] Ladon et al have shown an increase of both chromosome translocations and aneuploidy in peripheral blood lymphocytes at 6, 12, and 24 months after MOM-THA [63]. Therefore the association of increased chromium plasma levels and increased risk of mortality through cancer warrants further research. The arguments for this association are the carcinogenic effect of chromium through direct DNA-damage [56], strong relationship between whole blood levels of chromium and total chromosomal aberration indices in patients with MOM [62, 63], chronically increased chromium plasma levels in patients with MOM [6], and increased long-term mortality in MOM patients as shown by the present systematic review. Furthermore, patients with MOM THA are not only exposed to a single metal but to a "cocktail" of metal ions including chromium, cobalt, titanium, nickel, and molybdenum, of which at least two are potentially carcinogenic (chromium and nickel) and one is possibly carcinogenic (cobalt) [6, 56].

### Strengths and limitations

Our search strategy was thorough and complete. We included studies published between 1975 and 2014. Also, after contacting corresponding persons, we were able to include additional RCTs (both peer-reviewed papers and clinical trial reports) from trial registries such as clinicaltrials.org. In total, we were able to include 47 papers, including several with follow-up of 10 years or more.

For non-resurfacing THA, the surgical procedure is almost identical for MOM and non-MOM THA. Even the implants are identical with respect to the femoral stem and outer shell of the cup. The only difference is the bearing (liner and femoral head) that is inserted during the procedure. Therefore the surgical skill/experience is the same for non-resurfacing MOM and non-MOM THA.

The fact that the results from observational studies of first generation MOM THA concur with those from the RCTs reinforces the conclusion that MOM patients have an increased risk of mortality in the long run compared to non-MOM patients.

We should consider some limitations. Most RCTs had problems with allocation concealment and blinding during follow-up. However, the primary outcome of mortality is an objective outcome measure and is therefore very unlikely to be misclassified due to problems with blinding. Lack of blinding could have resulted in intensified follow-up for patients with MOM THA once the issues with MOM became apparent. However, none of the included studies mentioned differences in follow-up. Also, if we were to assume intensified follow-up (due to public awareness) for patients with MOM, and that this follow-up would be successful in reducing mortality and morbidity, these effects would have led to an underestimation of the observed effect on mortality and surgical morbidity (revisions) in MOM THA. Thus, in this case the increased risk of long term mortality for MOM THA and the increased risk of revision for MOM THA would even be higher. These unlikely effects would thus not change our conclusions.

There was limited data from RCTs on medical complications. Future RCTs and new reports of existing RCTs should therefore report these complications in a systematic way.

### Comparison with other studies

Visuri et al [3, 44] showed increased mortality and increased cancer incidence from MOM THA in an observational study of first generation MOM hip prostheses implanted between 1967 and 1973: the McKee-Farrar. This study is particularly interesting since the McKee-Farrar is part of the evolution of total hip prostheses and was not subject to modern marketing, nor was it labeled a "sports hip". The results from Visuri et al [3] are in accordance with the results of the RCTs of second generation (modern) MOM THA, therefore reinforcing our conclusion that MOM THA is associated with an increased risk of mortality in the long term.

Kendal et al found increased mortality in non-MOM THA in a registry based study of second generation MOM THA using propensity score matching [7]. Their results are in disagreement with the results of our meta-analysis, likely because registry-based studies are subject to residual confounding by indication. Indeed, Kandala et al have shown that confounding by indication is likely for the Kendal study, since one-fifth of the metal-on-metal subjects are predicted to live beyond 100 years of age, making metal-on-metal total hip replacement more beneficial for longevity than any other known treatment [9]. This latter finding is highly unlikely, and confounding by indication for the Kendal study is the most likely reason for this predicted longevity.

Mäkela et al found at short-term follow-up no difference in cancer incidence and cause-specific mortality in patients with second generation MOM THA compared to non-MOM THA [48]. For the short-term follow-up, their results are in agreement with the results of our meta-analysis.

### Conclusions and implications for clinicians and researchers

Studies with follow-up of greater than 10 years seem to suggest an increased risk of mortality in MOM THA compared to non-MOM THA. Additionally there is an increased risk of revision in MOM THA compared to non-MOM THA. In the light of these results, more long-term follow-up of RCTs reporting mortality is paramount. Also, future observational studies should address the dose-response association of person/hip years exposure to MOM THA and/or levels of metal ions to the risk of mortality and other medical complications e.g. cancer incidence, cardiomyopathy and renal failure.

There is currently no case for the use of MOM THA giving the increased risk of long-term mortality and revision without any proven major advantage. Considering the results discussed above, it is prudent to closely follow the patients that have already received a MOM THA, especially in the long-term.

## Supporting Information

S1 File Prospero Protocol. (PDF)

S2 File PRISMA Checklist. (PDF)

S3 File Search Strategy. (DOC)
