# Supplementary material for: Increased Mortality in Metal-on-Metal versus Non-Metal-on-Metal Primary Total Hip Arthroplasty at 10 Years and Longer Follow-Up: A Systematic Review and Meta-Analysis
Source: PLoS One. 2016 Jun 13;11(6):e0156051. doi: 10.1371/journal.pone.0156051 (PMC4905643; doi:10.1371/journal.pone.0156051)
Supplement: S1 File — (PDF) [file pone.0156051.s001.pdf]

PROSPERO International prospective register of systematic reviews

Metal on metal versus non metal on metal primary total hip arthroplasty: a systematic review and meta-analysis of randomized controlled trials

Bart Pijls, Jennifer Meessen, Jan Schoones, Huub van der Heide, Rob Nelissen

Citation

Bart Pijls, Jennifer Meessen, Jan Schoones, Huub van der Heide, Rob Nelissen. Metal on metal versus non metal on metal primary total hip arthroplasty: a systematic review and meta-analysis of randomized controlled trials. PROSPERO 2014:CRD42014007417 Available from
http://www.crd.york.ac.uk/PROSPERO_REBRANDING/display_record.asp?ID=CRD42014007417

Review question(s)

What is the mortality for metal on metal articulations compared to non metal on metal articulations after primary total hip arthroplasty in patients with endstage primary and secondary osteoarthritis?

What is the morbidity, expressed in surgical and medical complications, for metal on metal articulations compared to non metal on metal articulations after primary total hip arthroplasty in patients with endstage primary and secondary osteoarthritis?

Searches

The search strategy was devised in cooperation with a librarian experienced in the field of total hip arthroplasty. The following databases will be searched: PubMed, MEDLINE, EMBASE, Web of Science, Cochrane, CINAHL, Academic search premier. The following journal databases will be searched: ScienceDirect, Wiley-Blackwell.

References of included articles will be screened for relevant studies. Clinical trial registers will be searched to identify any ongoing trial or trials that have been completed but not yet published.

The search strategy consisted of the following components, each defined by a combination of controlled vocabulary and free text terms:

1) implant type: metal on metal, resurfacing and brand names

2) total hip arthroplasty

3) randomized controlled trial

All bibliographic records identified through the electronic searches will be collected in an electronic reference database.

Types of study to be included

Studies* will be subjected to the following inclusion and exclusion criteria:

Inclusion criteria:

1) primary total hip arthroplasty

2) comparison of metal on metal bearing with non metal on metal bearing

3) randomized controlled trial or quasi randomized controlled trial

4) Follow up of three months or more

Exclusion criteria are (during data extraction):

1) only bilateral cases with metal on metal and non metal in metal in the same patient, since this would not allow to determine mortality for the groups separately.

2) no reporting/evaluation of mortality of morbidity

*Full text studies, abstracts and reports from trial registers.

Condition or domain being studied
Endstage primary and secondary osteoarthritis.

Participants/ population
Patients treated with primary total hip arthroplasty, either metal on metal or non metal on metal, due to endstage primary and secondary osteoarthritis after failed conservative treatment.

Intervention(s), exposure(s)
Primary total hip arthroplasty with metal on metal bearings including resurfacing total hip with metal bearings.

Comparator(s)/ control
Primary total hip arthroplasty with non metal on metal bearings (e.g. metal on poly-ethylene, metal on ceramic, ceramic on ceramic, ceramic on poly-ethylene).

Outcome(s)
Primary outcomes
Mortality, expressed as number of patients who have died in the study period.

Secondary outcomes
Morbidity, expressed as number of surgical and medical complications.

Data extraction, (selection and coding)
Initial screening for eligibility on the basis of title and abstract will be performed by two reviewers independently with both recording their findings in a pre-designed electronic database. Both databases will be compared and any disagreements will be resolved by either consensus or by consulting a referee. When the information in the abstract does not suffice or in case of any doubt the studies will remain eligible.

The full text of eligible studies will be independently evaluated by two reviewers with both recording their findings in a pre-designed electronic database. Both databases will be compared and any disagreements will be resolved by either consensus or by consulting a referee. During both the eligibility phase and inclusion and exclusion phase reasons for exclusion will be documented. The study flow will be presented in a PRISMA diagram (Moher 2009).

Two reviewers will independently extract data from included studies regarding the outcomes (mortality and morbidity), patient demographics, study characteristics and implant specifications in a pre-defined electronic data sheet that will be designed during a trial data extraction on random sample of eligible studies. Both databases will be compared and any disagreements will be resolved by either consensus or by consulting a referee.

Risk of bias (quality) assessment
Risk of bias of the included studies will be appraised independently by two reviewers, at the level outcome using the CLEAR-NPT checklist (Boutron 2005) and Cochrane risk of bias table. The CLEAR-NPT checklist was specifically designed to appraise the methodological quality of non-pharmacological trials and contains among others items related to the standardization of the intervention, care provider influence, and additional measures to minimize the potential bias from lack of blinding of participants, care providers, and outcome assessors. Any disagreements will be resolved either by consensus or by consulting a referee.

Strategy for data synthesis
The data from the included RCTs will be pooled in a meta-analysis if there is sufficient similarity between studies

regarding methodological, clinical and implant characteristics. Data will be combined for meta-analysis with the random-effects model according to DerSimonian and Laird to determine the risk differences (RDs) and number needed to treat (NNT). Also the Odds Ratio (OR) will be determined with Peto's method, because studies with empty cells are expected (rare events).

The amount of statistical heterogeneity will be assessed through visual inspection of Forest plots and by calculating tau-squared statistics and I-squared statistics. In case of statistical heterogeneity and if data allow, potential sources of statistical heterogeneity will be explored through subgroup analysis (implant specifics (e.g. resurface / non-resurface metal on metal, type of non metal on metal bearing), primary / secondary osteoarthritis, items from CLEAR NPT/ Cochrane risk of bias tables) and with random effects meta-regression (e.g. duration of follow-up, age).

To assess for publication bias we will construct a funnel plot for studies reporting the primary outcome. In case of asymmetry in the funnel plot, or publication bias suspected based on the trial registries, a trim and fill method and cumulative meta-analyses will be used to explore the magnitude and direction of publication bias.

Analysis of subgroups or subsets

See also strategy for data synthesis above. Subgroup analyses will be performed if data permit on type of metal on metal bearing (resurfacing vs non resurfacing) type of non metal bearing (metal on poly-ethylene, metal on ceramic, ceramic on ceramic, ceramic on poly-ethylene), head size, fixation (cemented, cementless), indication (primary / secondary osteoarthritis) and methodological items from the CLEAR NPT and Cochrane risk of bias table. Furthermore the modifying effect of duration of follow-up and age will be explored with random effect meta-analyses. If considered appropriate sensitivity analyses will explore the effect of other non pre-defined items/factors. These will be labeled as "non pre-defined" in the results.

List of pre-defined factors:

type of metal bearing (resurfacing vs non resurfacing)

type of non-metal bearing

head size

fixation method (cemented, cementless)

indication for THA (primary vs secondary osteoarthritis)

methodological items from CLEAR NPT and Cochrane risk of bias table

duration of follow-up

mean age at operation

Gender distribution (% of females and males)

Pre-operative health (ASA scores)

Dissemination plans

The results of this review will be presented at national and international conferences and published in peer reviewed journals as appropriate.

Contact details for further information

Bart Pijls

Dept Orthopaedics

LUMC (Leiden University Medical Center)

Postbus 9600

2300 RC Leiden

Netherlands

b.g.c.w.pijls@lumc.nl

Organisational affiliation of the review
Leiden University Medical Center, Dept of Orthopaedics

https://www.lumc.nl/con/2020/82590/

Review team
Dr Bart Pijls, Leiden University Medical Center, Dept of Orthopaedics
Ms Jennifer Meessen, Leiden University Medical Center, Dept of Orthopaedics
Mr Jan Schoones, Leiden University Medical Center, Walaeus Library
Dr Huub van der Heide, Leiden University Medical Center, Dept of Orthopaedics
Professor Rob Nelissen, Leiden University Medical Center, Dept of Orthopaedics

Anticipated or actual start date
14 January 2014

Anticipated completion date
01 July 2014

Funding sources/sponsors
Not applicable.

Conflicts of interest
None known

Language
English

Country
Netherlands

Subject index terms status
Subject indexing assigned by CRD

Subject index terms
Arthroplasty, Replacement, Hip; Hip Prosthesis; Humans; Metal-on-Metal Joint Prostheses; Osteoarthritis, Hip

Reference and/or URL for protocol
http://www.crd.york.ac.uk/PROSPEROFILES/7417_PROTOCOL_20140101.pdf

Stage of review
Ongoing

Date of registration in PROSPERO
03 February 2014

Date of publication of this revision
29 July 2014

DOI
10.15124/CRD42014007417

Stage of review at time of this submission	Started	Completed
Preliminary searches	No	Yes
Piloting of the study selection process	No	Yes
Formal screening of search results against eligibility criteria	No	Yes
Data extraction	No	Yes
Risk of bias (quality) assessment	No	Yes
Data analysis	No	Yes

PROSPERO
International prospective register of systematic reviews
The information in this record has been provided by the named contact for this review. CRD has accepted this information in good faith and registered the review in PROSPERO. CRD bears no responsibility or liability for the content of this registration record, any associated files or external websites.
