# Supplementary material for: Increased Mortality in Metal-on-Metal versus Non-Metal-on-Metal Primary Total Hip Arthroplasty at 10 Years and Longer Follow-Up: A Systematic Review and Meta-Analysis
Source: PLoS One. 2016 Jun 13;11(6):e0156051. doi: 10.1371/journal.pone.0156051 (PMC4905643; doi:10.1371/journal.pone.0156051)
Supplement: S3 File — (DOC) [file pone.0156051.s003.doc]

Search strategy for Pubmed for RCTs:

(ASR[tw] OR DePuy[tw] OR BHR[tw] OR Finsbury[tw] OR "Smith and Nephew"[tw] OR Conserve Plus[tw] OR Wright Medical Technology[tw] OR Cormet[tw] OR cormet 2000[tw] OR Corin[tw] OR Durom[tw] OR Zimmer[tw] OR McMinn[tw] OR Corin Group[tw] OR Accis[tw] OR Implantcast[tw] OR Adept[tw] OR Eska-Bionik[tw] OR Eska[tw] OR Icon[tw] OR "International Orthopaedics"[tw] OR Mitch[tw] OR Stryker[tw] OR Recap[tw] OR Biomet[tw] OR Minimally Invasive Hip Resurfacing[tw] OR Comis Orthopaedics[tw] OR Comis[tw] OR BMHR[tw] OR Birmingham[tiab] OR Mid-Head Resection[tw] OR "Smith & Nephew"[tw] OR metasul[tw] OR zimmer[tw] OR Ring[tw] OR Stanmore[tw] OR Muller[tw] OR mckeefarrar[tw] OR "mckee farrar"[tw] OR "mckeefarrar"[tw] OR "birmingham hip"[all fields] OR "birmingham hips"[all fields] OR "BHR"[tw] OR "articular surface replacement"[all fields] OR "ASR"[tw] OR "comet"[tw] OR "Metal-on-Metal Joint Prostheses"[Mesh] OR "MoM"[tw] OR metalonmetal*[all fields] OR "metal-on-metal"[all fields] OR "metal on metal"[all fields] OR "resurface"[all fields] OR "resurfacing"[all fields] OR resurfac*[all fields]) AND (tha[tw] OR "total hip"[All Fields] OR "total hips"[All Fields] OR (("total joint"[All Fields] OR "total joints"[All Fields]) AND ("hip"[MeSH Terms] OR "hip"[All Fields] OR "hips"[All Fields])) OR ((total[All Fields] AND ("hip"[MeSH Terms] OR "hip"[All Fields])) AND (("prostheses and implants"[MeSH Terms] OR "prostheses"[All Fields] OR "implants"[All Fields] OR "prosthesis"[All Fields] OR "implant"[All Fields] OR prosthetic[All Fields] OR endoprosthesis[All Fields] OR endoprostheses[All Fields] OR endoprosthetic[All Fields] OR "arthroplasty"[MeSH Terms] OR "arthroplasty"[All Fields] OR arthroplast*[All Fields] OR replacement[tiab])) OR "arthroplasty, replacement, hip"[MeSH Terms] OR ("arthroplasty"[All Fields] AND "replacement"[All Fields] AND "hip"[All Fields]) OR "hip replacement arthroplasty"[All Fields] OR "arthroplasty, replacement, hip"[MeSH Terms] OR ("arthroplasty"[All Fields] AND "replacement"[All Fields] AND "hip"[All Fields]) OR "hip replacement arthroplasty"[All Fields] OR (("hip"[MeSH Terms] OR "hip"[All Fields]) AND ("arthroplasty"[MeSH Terms] OR "arthroplasty"[All Fields])) OR ("hip"[All Fields] AND "replacement"[All Fields]) OR "hip replacement"[All Fields]) OR "hip prosthesis"[MeSH Terms] OR ("hip"[All Fields] AND "prosthesis"[All Fields]) OR "hip prostheses"[All Fields]) AND (RCT[all fields] OR RCTS[all fields] OR "random"[all fields] OR random*[all fields] OR "randomised"[all fields] OR "randomised"[all fields] OR "Randomised Controlled Trial"[Publication Type] OR "Double-Blind Method"[mesh] OR "Single-Blind Method"[mesh] OR "Random Allocation"[mesh] OR "Controlled Clinical Trial"[Publication Type])

Search strategy for Pubmed for observational studies:

(ASR[tw] OR DePuy[tw] OR BHR[tw] OR Finsbury[tw] OR "Smith and Nephew"[tw] OR Conserve Plus[tw] OR Wright Medical Technology[tw] OR Cormet[tw] OR cormet 2000[tw] OR Corin[tw] OR Durom[tw] OR Zimmer[tw] OR McMinn[tw] OR Corin Group[tw] OR Accis[tw] OR Implantcast[tw] OR Adept[tw] OR Eska-Bionik[tw] OR Eska[tw] OR Icon[tw] OR "International Orthopaedics"[tw] OR Mitch[tw] OR Stryker[tw] OR Recap[tw] OR Biomet[tw] OR Minimally Invasive Hip Resurfacing[tw] OR Comis Orthopaedics[tw] OR Comis[tw] OR BMHR[tw] OR Birmingham[tiab] OR Mid-Head Resection[tw] OR "Smith & Nephew"[tw] OR metasul[tw] OR zimmer[tw] OR Ring[tw] OR Stanmore[tw] OR Muller[tw] OR mckeefarrar[tw] OR "mckee farrar"[tw] OR "mckeefarrar"[tw] OR "birmingham hip"[all fields] OR "birmingham hips"[all fields] OR "BHR"[tw] OR "articular surface replacement"[all fields] OR "ASR"[tw] OR "comet"[tw] OR "Metal-on-Metal Joint Prostheses"[Mesh] OR "MoM"[tw] OR metalonmetal*[all fields] OR "metal-on-metal"[all fields] OR "metal on metal"[all fields] OR "resurface"[all fields] OR "resurfacing"[all fields] OR resurfac*[all fields]) AND (tha[tw] OR "total hip"[All Fields] OR "total hips"[All Fields] OR (("total joint"[All Fields] OR "total joints"[All Fields]) AND ("hip"[MeSH Terms] OR "hip"[All Fields] OR "hips"[All Fields])) OR ((total[All Fields] AND ("hip"[MeSH Terms] OR "hip"[All Fields])) AND (("prostheses and implants"[MeSH Terms] OR "prostheses"[All Fields] OR "implants"[All Fields] OR "prosthesis"[All Fields] OR "implant"[All Fields] OR prosthetic[All Fields] OR endoprosthesis[All Fields] OR endoprostheses[All Fields] OR endoprosthetic[All Fields] OR "arthroplasty"[MeSH Terms] OR "arthroplasty"[All Fields] OR arthroplast*[All Fields] OR replacement[tiab])) OR "arthroplasty, replacement, hip"[MeSH Terms] OR ("arthroplasty"[All Fields] AND "replacement"[All Fields] AND "hip"[All Fields]) OR "hip replacement arthroplasty"[All Fields] OR "arthroplasty, replacement, hip"[MeSH Terms] OR ("arthroplasty"[All Fields] AND "replacement"[All Fields] AND "hip"[All Fields]) OR "hip replacement arthroplasty"[All Fields] OR (("hip"[MeSH Terms] OR "hip"[All Fields]) AND ("arthroplasty"[MeSH Terms] OR "arthroplasty"[All Fields])) OR ("hip"[All Fields] AND "replacement"[All Fields]) OR "hip replacement"[All Fields]) OR "hip prosthesis"[MeSH Terms] OR ("hip"[All Fields] AND "prosthesis"[All Fields]) OR "hip prostheses"[All Fields]) AND ("Neoplasms/etiology"[mesh] OR "mortality"[Subheading] OR "mortality"[tw] OR "mortality"[mesh] OR "Cause of Death"[mesh] OR "Neoplasms/epidemiology"[majr] OR "Osteoarthritis, Hip/mortality"[mesh] OR "Cardiomyopathies"[Mesh] OR "Cardiomyopathies"[tw] OR "Cardiomyopathy"[tw] OR "Renal Insufficiency"[Mesh] OR "renal failure"[tw] OR "kidney failure"[tw] OR "Observational Study" [Publication Type] OR "Observational Study"[tw] OR "Observational Studies"[tw] OR "Observational Study as Topic"[Mesh])
